# Recent advances in small molecule dye-based nanotheranostics for NIR-II photoacoustic imaging-guided cancer therapy

**DOI:** 10.3389/fbioe.2022.1002006

**Published:** 2022-09-29

**Authors:** Wen Zhou, Likun Yin, Xuheng Zhang, Tingting Liang, Zixin Guo, Yaxin Liu, Chen Xie, Quli Fan

**Affiliations:** State Key Laboratory for Organic Electronics and Information Displays & Institute of Advanced Materials IAM, Nanjing University of Posts & Telecommunications, Nanjing, China

**Keywords:** NIR-II photoacoustic imaging, small molecule dye, phototheranostics, combination therapy, tumor imaging

## Abstract

Photoacoustic (PA) imaging in the second near-infrared (NIR-II) window has gained more and more attention in recent years and showed great potential in the field of bioimaging. Until now, numerous materials have been developed as contrast agents for NIR-II PA imaging. Among them, small molecule dyes hold unique advantages such as definite structures and capability of fast clearance from body. By virtue of these advantages, small molecule dyes-constructed nanoparticles have relatively small size and show promise in the clinical translation. Thus, in this minireview, we summarize recent advances in small molecule dyes-based nanotheranostics for NIR-II PA imaging and cancer therapy. Studies about NIR-II PA imaging-guided phototherapy are first introduced. Then, NIR-II PA imaging-guided phototherapy-based combination therapeutic systems are reviewed. Finally, the conclusion and perspectives of this field are summarized and discussed.

## 1 Introduction

Photoacoustic (PA) imaging is a kind of optical imaging modality which utilizes light as the excitation source and collect ultrasound waves as the signal source (Attia et al., 2019; Yang et al., 2009). By virtue of such feature, PA imaging has both higher tissue penetration depth and spatial resolution (Hoelen et al., 1998; Wang and Hu, 2012). Until now, PA imaging has been applied for clinical detection of breast cancer, and shows a higher sensitivity than conventional ultrasound imaging (Valluru and Willmann, 2016). In addition, PA imaging can be facilely combined with ultrasound imaging to develop multimodal imaging modality as they both detect acoustic waves (Manwar et al., 2020). Although the great potential of PA imaging, only a few endogenous biomasses such as hemoglobin and deoxyhemoglobin show PA signals under laser irradiation, making it necessary to develop exogenous contrast agents for PA imaging to achieve a better imaging effect (Cheng et al., 2020; Cui et al., 2019; Huang and Pu, 2020; Weber et al., 2016).

Until now, variety of materials have been designed as PA contrast agents. For example, inorganic nanomaterials including gold nanoparticles (Kim et al., 2018; Mallidi et al., 2009), quantum dots (Guo et al., 2021; Lv et al., 2016), 2D materials (Yu et al., 2019) have been widely applied for PA imaging-related biomedical applications owing to their broad absorption spectra and high photothermal conversion efficiency (PCE). Apart from inorganic nanomaterials, organic materials have attracted more and more attention in phototheranostics (Zhang et al., 2022; Zhang et al., 2021). Two major kinds of organic materials, semiconducting polymers (SPs) and small molecule dyes, can both be developed into PA contrast agents (Chen et al., 2019; He et al., 2020a; Li et al., 2020; Xu and Pu, 2021; Zhang et al., 2019a). Owing to the advantages of high absorption coefficient and good photostability, SPs usually exhibit satisfactory PA signals (Pu et al., 2014; Sun et al., 2018; Chen et al., 2020a; Zhen et al., 2021). However, because of their high molecular weight, SPs are hardly cleared from body in a relatively short time, which may cause long-term toxicity (Gupta et al., 2021). In contrast, small molecule dyes have much lower molecular weights with definite structures. Such feature endows them with possibility of fast clearance from body, which shows great promise in clinical translation (Huang et al., 2019a; Zhen et al., 2018). For example, indocyanine green (ICG) has been approved by FDA as optical imaging contrast agent for human body (He et al., 2020b; Soni et al., 2016). However, the photostability of ICG and its derivatives are poor, limiting their further applications (Noh et al., 2011). Thus, designing novel small molecule dyes as PA contrast agents is of great significance.

Although PA imaging has a relatively deep tissue penetration among all the optical imaging modalities, the penetration depth is still restricted compared with traditional modalities such as CT and MRI (Penet et al., 2010; Zhou et al., 2020). Most PA imaging-based theranostics utilize first near-infrared window (NIR-I, 700–900 nm) light as excitation source. Compared with NIR-I light, second near-infrared window (NIR-II, 1,000–1700 nm) light shows both lower tissue absorption and scattering, resulting in a higher tissue penetration depth (Chen et al., 2020b; Zhao et al., 2018). Thus, using NIR-II light as excitation source of PA imaging can achieve a better tissue penetration and imaging effect. Compared with NIR-I PA contrast agents, NIR-II PA contrast agents have longer absorption wavelengths (Miao and Pu, 2018; Yin et al., 2021a). Until now, variety of inorganic and SPs-based nanomaterials have been reported for NIR-II PA imaging and phototheranostics (Cao et al., 2018; Jiang et al., 2019; Wu et al., 2017; Zhang et al., 2019b). However, for small molecule dyes, NIR-II absorbing molecules usually have larger π-conjugated backbones and more complicated structures than NIR-I absorbing molecules, which makes it more difficult to be synthesized (Antaris et al., 2016; Li et al., 2022; Wang et al., 2019).

By virtue of the great potential of NIR-II PA imaging, several excellent reviews have summarized the progress in this field (Zhen et al., 2021; Yin et al., 2021a; Li et al., 2022; Upputuri and Pramanik, 2019; Huang et al., 2019b). However, most of them focused on the PA imaging systems or contrast agents such as inorganic nanomaterials or semiconducting polymer nanoparticles (SPNs). Inspired by the significant advantages of small molecule dyes, we summarize recent advances of small molecule dyes in NIR-II PA imaging-related phototheranostics in this minireview. In the following, we first introduce the design and classification of NIR-II PA contrast agents. Then, the application of small molecule dyes for NIR-II PA imaging-guided phototheranostics and combination therapy are sequentially discussed. Finally, the conclusion and future perspectives of this field are given.

## 2 Design and classification of NIR-II PA contrast agents

The reported small molecule-based NIR-II PA contrast agents could be generally classified into three types: NIR-II-absorbing molecules, metal complexes and charge-transfer complexes. NIR-II-absorbing molecules were the most reported one for NIR-II PA imaging. Until now, several kinds of NIR-II-absorbing molecules were designed for NIR-II phototheranostics, such as donor-acceptor-donor (D-A-D) type oligomers, cyanine and boron-dipyrromethenes (BODIPY) derivatives (Yin et al., 2021b). Cyanine and BODIPY derivatives have been developed into NIR-II PA contrast agents, which introduced in the following sections, and almost all of them showed a red-shifted absorption after being encapsulated into nanoparticles. Such feature endowed them with unique advantages for NIR-II PA imaging. Metal complexes were prepared by chelating metal ions such as Fe and Cu with small molecule dyes (Chalati et al., 2011; Meng et al., 2015) Due to the ligand to metal charge transfer (LMCT) effect which was widely reported in metal organic frameworks (MOFs), metal complexes had very broad absorption which could tail to NIR-II region, although their maximum absorption may not locate in NIR-II region (Zhao et al., 2021). This made them suitable for NIR-II PA imaging. Charge transfer complexes were formed between electron-donating and electron-accepting molecules. The stronger donating or accepting molecules used, the molecules with longer absorption would obtain (Zhang et al., 2017). Charger transfer complexes may have a much longer absorption than other two kinds of small molecule-based NIR-II PA contrast agents (Table 1).

**TABLE 1:** Summary of small molecule-based contrast agents for NIR-II PA imaging.

Type	Name	Amphiphilic molecules	Maximum absorption wavelength	PCE	Ref
NIR-II absorbing molecule	IR 1048	DPPC/DOPC/DSPC	1,100 nm	41.17%	Geng et al. (2020)
	CyFaP	F127	1,040 nm	N.A.	Chitgupi et al. (2019)
	HBP	F127	1,012 nm	50.1%	Zhang et al. (2020)
Metal complex	BDP-Fe	F127	660 nm	49%	Ou et al. (2020)
Charge-transfer complex	TMB-F4TCNQ	F127	>1,300 nm	42.4%	Ou et al. (2021)

N.A.: not available, DPPC: 1,2-dipalmitoyl-*sn*-glycero-3-phosphocho-line, DOPC: 1,2-dioleoyl-*sn*-glycero-3-phosphocholine, DSPC: 1,2-distearoyl-sn-glycero-3-phosphocholine.

All of these molecules or complexes mentioned had poor water solubility and required the addition of amphiphilic molecules to increase their solubility in water. F127 was the mostly chosen amphiphilic molecules, not only because its commercial availability and low cost, but also the feasibility of preparing high concentration nanoparticles *via* surfactant-stripped approach (Zhang et al., 2018). These molecules or complexes had satisfactory PCE (higher than 40%), making them good candidates for NIR-II PA imaging-guided photothermal therapy (PTT).

## 3 NIR-II PA imaging-guided phototheranostics

**FIGURE 1:**
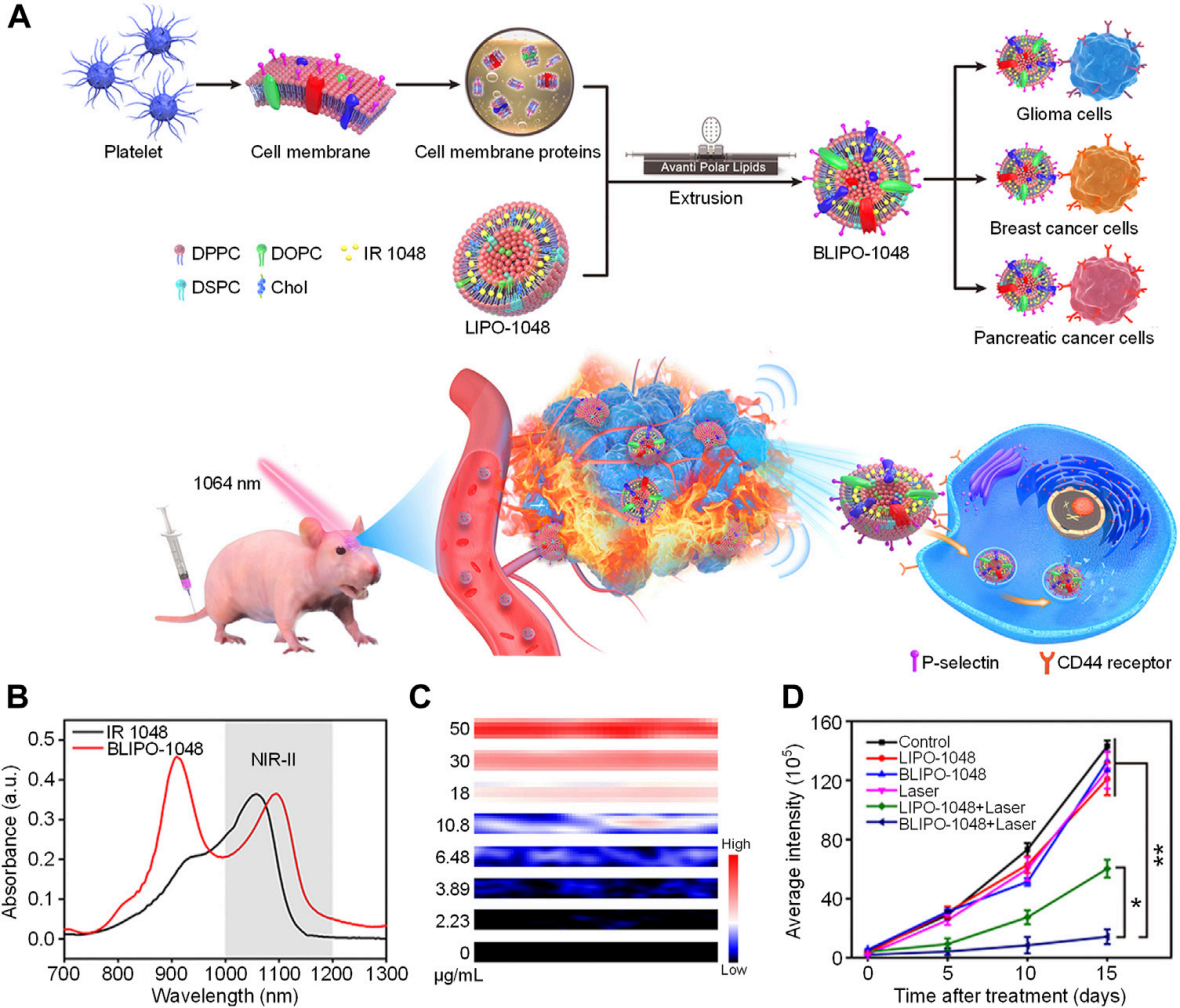
**(A)** Schematic illustration of preparation of BLIPO-1048 for NIR-II phototheranostics. **(B)** Absorption spectra of IR 1048 and BLIPO-1048. **(C)** NIR-II PA images of BLIPO-1048 under different concentrations. **(D)** Semiquantitative bioluminescence intensity in the brain of mice. The error bars represent standard deviations of six separate measurements (*n* = 6). *p < 0.05, **p < 0.01. Adapted from (Geng et al., 2020). Copyright^©^ 2020 American Chemical Society.

Phototheranostics which combine optical imaging and phototherapy into one system have attracted tremendous attention in recent years (Cai et al., 2018). However, most phototheranostic nanosystems utilize NIR-I light as the source for optical imaging and phototherapy (Li et al., 2018a; Wan et al., 2020) The low penetration depth of NIR-I light significantly restricts the theranostic efficacy. To address such issue, variety of NIR-II light-mediated phototheranotic systems have been designed and developed (Dai et al., 2021; Yin et al., 2020). Zheng et al. developed a platelet-camouflaged nanoprobe (BLIPO-1048) for tumor active-targeting NIR-II PA imaging-guided PTT (Geng et al., 2020). A commercially available NIR-II-absorbing small molecule dye IR 1048 was first encapsulated by amphiphilic lipid molecules to give IR 1048-loaded liposome (LIPO-1048). The membrane proteins of platelets were then extracted and coated onto the surface of LIPO-1048 *via* mechanical extrusion treatments to obtain BLIPO-1048. After i. v. Injection of BLIPO-1048 into tumor bearing mice, the nanoprobes could accumulate into tumor site *via* active targeting owing to the membrane proteins on their surface. Under 1,064 nm laser irradiation, BLIPO-1048 could generate NIR-II PA signal to delineate the tumor site. Meanwhile, the NIR-II photothermal effect of BLIPO-1048 may kill tumor cells (Figure 1A).

BLIPO-1048 had a NIR-II absorption peak at near 1,100 nm, which showed an obvious red-shift compared with IR 1048 (Figure 1B). In addition, the fluorescence intensity of BLIPO-1048 had a 39% decrease compared with IR 1048. These results could be attributed to the aggregation of IR 1048 within BLIPO-1048. Under 1,064 nm laser irradiation, the temperature of BLIPO-1048 solution increased more than 30°C, and its PCE was calculated as 41.17%. Meanwhile, BLIPO-1048 showed strong PA intensity at 1,064 nm. With the increase of concentration, its NIR-II PA intensity increased linearly, demonstrating the feasibility of quantitative study (Figure 1C). By virtue of the membrane protein CD47 on the surface of BLIPO-1048, BLIPO-1048 had a lower uptake by macrophages than LIPO-1048 without cell membrane on the surface. In contrast, BLIPO-1048 showed high tumor cell uptake, which could be attributed to the CD44 protein on its surface. Without laser irradiation, BLIPO-1048 showed good cytocompatibility towards both C6 glioma and bEnd.3 cells. Under 1,064 nm laser irradiation, both LIPO-1048 and BLIPO-1048 showed cytotoxicity against tumor cells. Compared with LIPO-1048 without cell membrane, BLIPO-1048 had a lower IC50 value under 1,064 nm laser irradiation, which could be ascribed to the higher cellular uptake of BLIPO-1048. The *in vivo* NIR-II PA imaging experiments were conducted by using three different tumor models (pancreatic, breast and glioma tumor model). For all the models, BLIPO-1048 could effectively accumulate into tumor sites and showed stronger NIR-II PA signals than LIPO-1048. In addition, PTT-mediated by BLIPO-1048 showed the highest tumor inhibition rate (85.2%) among all the treatments (Figure 1D). These data indicated that the cell membrane proteins on the surface of BLIPO-1048 may enhance the tumor accumulation of nanoparticles and their PTT efficacy.

**FIGURE 2:**
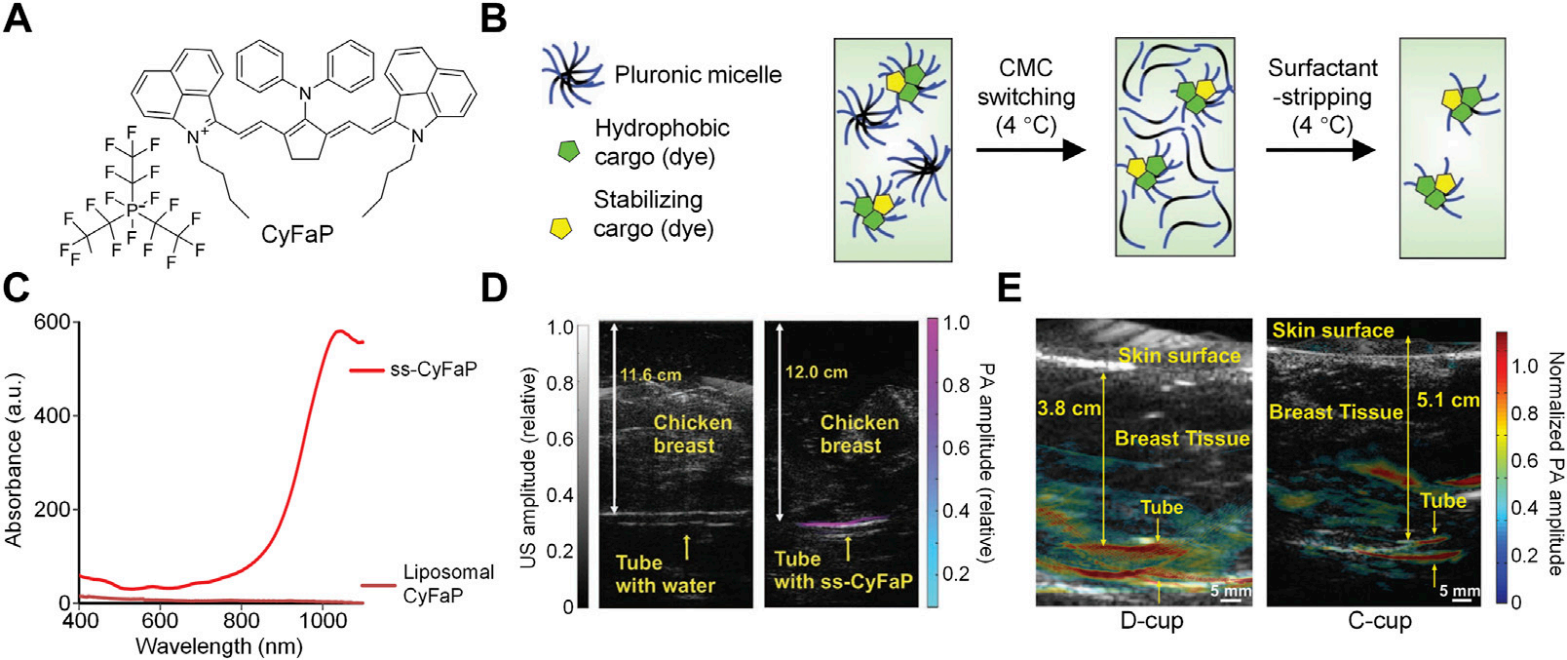
**(A)** Chemical structure of CyFaP. **(B)** Schematic illustration of preparation of surfactant-stripped micelles. **(C)** Absorption spectra of ss-CyFaP and Liposomal CyFaP. **(D)** PA overlaid with ultrasound images of tube with water or ss-CyFaP. **(E)** PA overlaid with ultrasound images of tube with ss-CyFaP under the breast of adult female volunteers. Adapted from (Chitgupi et al., 2019). Copyright^©^ 2019 Wiley-VCH Verlag GmbH & Co. KGaA, Weinheim.

As most NIR-II absorbing molecules are hydrophobic, it is necessary to encapsulate them into amphiphilic copolymers *via* nanoprecipitation method to form water-dispersible nanoparticles (Li and Pu, 2019; Yin et al., 2017). To prepare micelles with highly concentrated hydrophobic cargos, Lovell et al. developed a surfactant stripping method which could effectively remove excess amphiphilic copolymer F127 (Zhang et al., 2016a; Zhang et al., 2016b). By using similar method, they designed a NIR-II absorbing nanoprobe with extremely high concentration for NIR-II PA imaging (Chitgupi et al., 2019). A commercially available NIR-II absorbing dye CyFaP (Figure 2A) was co-loaded with Coenzyme Q10 (CoQ) into F127. To remove the free F127 and empty micelles, the solution was kept at 4°C so that F127 could well dissolved in the water and be removed *via* ultracentrifugation. After ultracentrifugation, the free F127 was removed from the solution, remaining the micelles with cargos loading. Such process was the so-called “surfactant stripping” which could obtain micelles with high concentration of cargos (Figure 2B). The obtained surfactant-stripped CyFaP micelles (ss-CyFaP) showed a much higher NIR-II absorption compared with CyFaP liposomes prepared by conventional method (Figure 2C). Only less than 5% CyFaP was precipitated after storage for 4 weeks, indicating the superior stability of ss-CyFaP.

The capability of ss-CyFaP for NIR-II PA imaging was evaluated. Even under the coverage of 12 cm chicken breast tissue, the NIR-II PA signal of ss-CyFaP solution could be clearly detected, and its signal-to-noise ratio (SNR) was as high as 24.3 dB. In contrast, no NIR-II PA signal was detected for water under the same condition (Figure 2D). Owing to the high penetration depth, ss-CyFaP was applied for *in vivo* PA imaging of tumor. Before injection of ss-CyFaP, almost no NIR-II PA signal was observed in the tumor region, while the signal increased significantly after i. v. Injection of ss-CyFaP for 6 h, which showed good tumor accumulation capability of ss-CyFaP. The potential clinical application of ss-CyFaP was demonstrated by imaging of ss-CyFaP through whole human breast. The NIR-II PA signal of ss-CyFaP solution could effectively penetrate 5.1 cm of human breast, indicating the promise of ss-CyFaP in human breast cancer detection (Figure 2E).

In summary, the above two studies showed the promise of small molecule dyes for NIR-II PA imaging and phototherapy. Both BLIPO-1048 and ss-CyFaP had strong NIR-II absorption and their NIR-II PA signals could have a penetration depth up to several centimeters. In addition, they both chose commercially available dyes coated with biocompatible cell membranes or FDA-approved amphiphilic materials, and the preparations of these nanosystems were simple enough. From a biological application point of view, these features made them great promise in clinical translation, which was confirmed by the fact that the NIR-II PA signal of ss-CyFaP could penetrate the whole human breast.

## 4 NIR-II PA imaging-guided combination therapy

Although phototherapy has shown great promise in the cancer therapy, its therapeutic efficacy is still limited (Torres et al., 2021; Xie et al., 2020a). For example, high temperature may induce the overexpression of heat shock proteins (HSPs) which can alleviate the damage of heat to cancer cells (Gao et al., 2020). On the other hand, photodynamic therapy (PDT) usually requires oxygen to generate reactive oxygen species (ROS) for cancer cell killing. However, the hypoxic environment of solid tumor may not provide enough oxygen (Li et al., 2018b). Thus, more and more studies have developed combination therapeutic nanosystems which combine phototherapy with other therapeutic modalities (Zhao et al., 2020; He et al., 2022). Some inhibitors or chemotherapy drugs were reported to suppress the HSP expression, by introducing them into PTT system, photothermal/chemo combination therapy can be achieved (Gao et al., 2021). HSPs may also be damaged by alkyl radicals (Wang et al., 2022). Thus, combining PTT with thermodynamic therapy can achieve synergistic therapeutic efficacy. In addition, both PTT and PDT are able to trigger immunogenic cell death (ICD) for immunotherapy. Such process is the so-called photo-immunotherapy which is a combination of phototherapy and immunotherapy (Wang et al., 2021). Overall, these additional therapeutic modalities can remedy the disadvantages of phototherapy and provide a better therapeutic efficacy for combination therapeutic system (Li et al., 2019).

**FIGURE 3:**
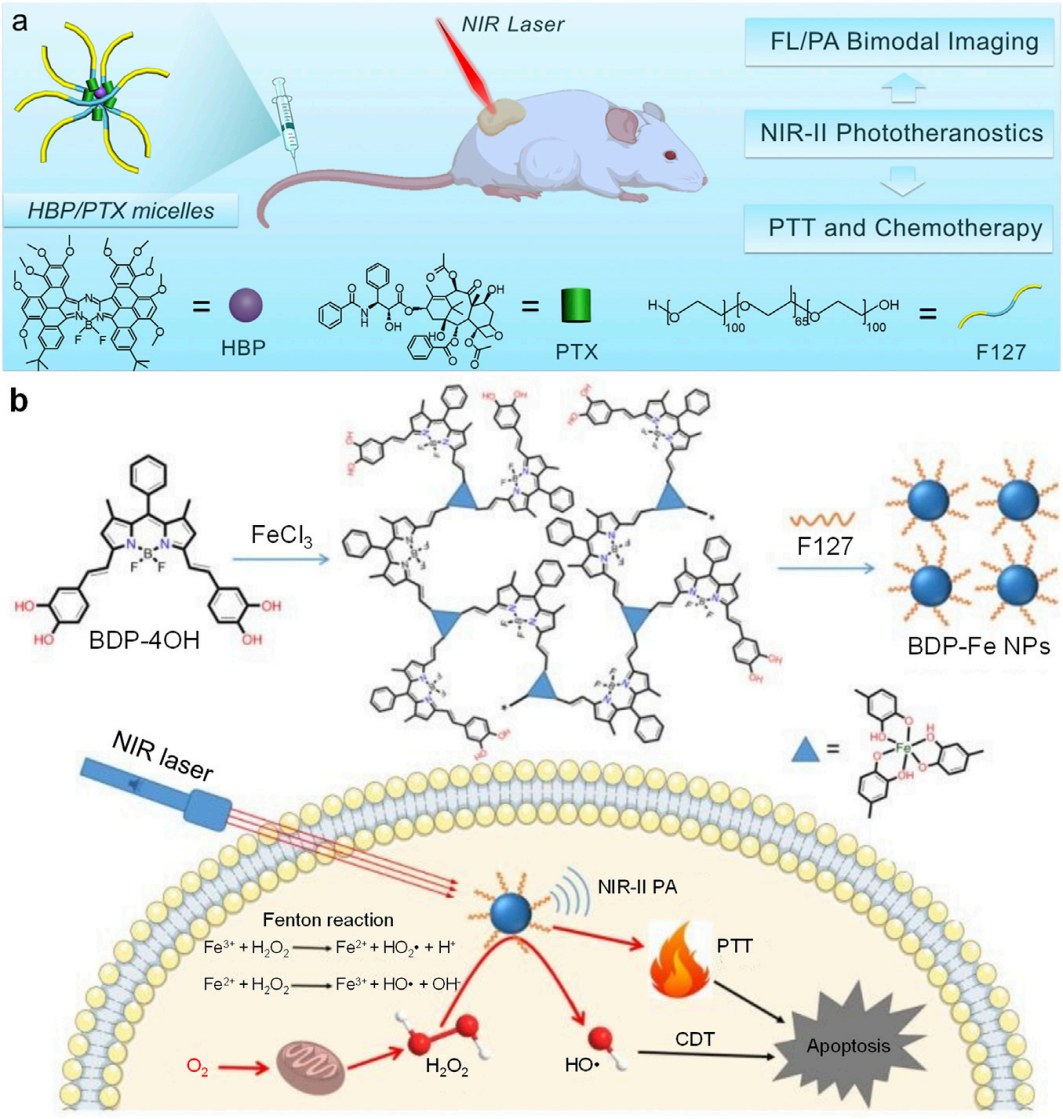
**(A)** Schematic illustration of structures of HBP/PTX micelles and their all-in-one NIR-II phototherapeutics for NIR-II fluorescence/PA imaging-guided PTT and chemotherapy. **(B)** Schematic illustration of preparation of BDP-Fe NPs and their applications for NIR-II PA imaging-guided PTT and CDT. Adapted from (Zhang et al., 2020; Ou et al., 2020). Copyright^©^ 2020 Elsevier B.V., 2020 Royal Society of Chemistry.

Feng et al. designed a surfactant-stripped micelles (HBP/PTX micelles) for NIR-II PA imaging-guided PTT/chemotherapy combination therapy (Zhang et al., 2020). HBP/PTX micelles were prepared by encapsulating a boron-dipyrromethenes (BODIPY) derivative (HBP) and paclitaxel (PTX) into F127, then removing excess F127 *via* surfactant stripping method (Figure 3A). Such micelles exhibited strong NIR-II absorption at 1,012 nm with NIR-II fluorescence emission. Under 1,064 nm laser irradiation, HBP/PTX micelles showed satisfactory photothermal effect which the solution temperature could increase up to 26 °C. HBP/PTX micelles could light up lymph nodes of mice by their NIR-II fluorescence signal. In addition, such micelles had high tumor accumulation and could delineate tumor tissue *via* NIR-II fluorescence imaging. HBP/PTX micelles showed strong NIR-II PA signal at 1,064 nm, which could be attributed to the encapsulated HBP molecule in the micelles. Similar with NIR-II fluorescence imaging, the NIR-II PA signal of HBP/PTX micelles could effectively detect the tumor of mice. HBP/PTX micelles also showed good anticancer efficacy. PTT could be induced by 1,064 nm laser irradiation, and the loaded PTX in the micelles was able to kill cancer cells by chemotherapy. Under laser irradiation, HBP/PTX micelles could totally inhibit the tumor growth of mice, showing excellent *in vivo* anticancer efficacy.

Chemodynamic therapy (CDT) is a kind of novel cancer therapeutic modality which catalyzes H_2_O_2_ in the tumor site into highly toxic ROS to kill cancer cells (Tang et al., 2019; Lin et al., 2018). Dong et al. synthesized a BODIPY derivative (BDP-4OH) with two catechol groups (Ou et al., 2020). BDP-4OH could chelate Fe^3+^ ion by its catechol groups and form three-dimensional compounds (BDP-Fe). After encapsulating such compounds by F127, the Fe-chelated BODIPY nanoparticles (BDP-Fe NPs) were obtained. BDP-Fe NPs could not only apply for NIR-II PA imaging, but also kill cancer cells *via* PTT/CDT combination therapy (Figure 3B). BDP-Fe NPs had broad absorption spectrum with strong absorption at 1,064 nm. Under neutral pH, BDP-Fe NPs could not catalyze H_2_O_2_ into hydroxyl radical. In contrast, hydroxyl radical was generated under pH 5.0 under catalysis of BDP-Fe NPs, triggering the process of CDT. Such result indicated that the CDT process could only be activated under acidic condition. BDP-Fe NPs also exhibited good photothermal effect, the temperature of BDP-Fe NPs solution could increase 23°C under 730 nm laser irradiation. Owing to the effect of PTT and CDT for BDP-Fe NPs, they were applied for *in vitro* and *in vivo* anticancer study. Under laser irradiation, the IC_50_ of HeLa cells could reach as low as 19.4 μg/ml with the treatment of H_2_O_2_. The confocal fluorescence imaging indicated that BDP-Fe NPs may increase the intracellular ROS level, showing that CDT process was triggered within HeLa cells. Because of the relatively strong NIR-II absorption, BDP-Fe NPs were applied for NIR-II PA imaging of tumor. After i. v. Injection of BDP-Fe NPs, the NIR-II PA signal in the tumor site increased gradually, demonstrating the accumulation of BDP-Fe NPs in the tumor and the capability of *in vivo* NIR-II PA imaging of BDP-Fe NPs. Under 730 nm laser, BDP-Fe NPs could effectively inhibit the growth of tumor, proving the anticancer efficacy of PTT/CDT combination therapy induced by BDP-Fe NPs.

**FIGURE 4:**
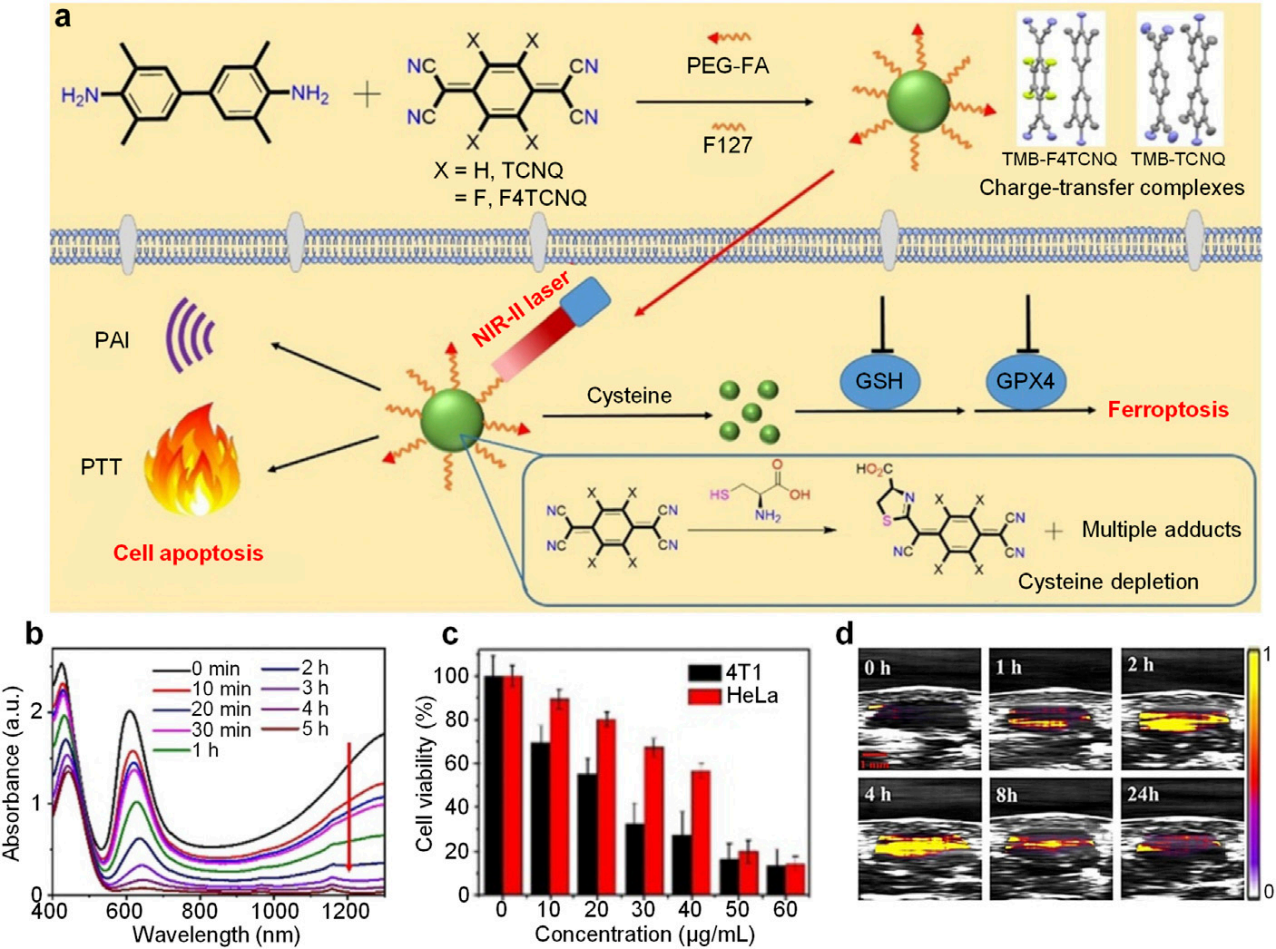
**(A)** Schematic illustration of preparation of charge-transfer complex nanoparticles and their applications in NIR-II PA imaging, PTT and ferroptosis. **(B)** Absorption spectrum change of TMB-F4TCNQ under treatment of cysteine with time at 50°C. **(C)** Viability of 4T1 or HeLa cells treated with different concentrations of TMB-F4TCNQ under 1,060 nm laser irradiation. **(D)** NIR-II PA images of tumor region from TMB-F4TCNQ-treated 4T1 tumor-bearing mice at different post-injection time. The error bars represent standard deviations of three separate measurements (*n* = 3). Adapted from (Ou et al., 2021). Copyright^©^ 2021 Wiley-VCH GmbH.

Glutathione (GSH) is commonly overexpressed in cancer cells which plays important role in regulating the intracellular ROS level, and depleting GSH may induce ROS-mediated ferroptosis of cancer cells (Sang et al., 2020). Cysteine is a critical precursor in the biosynthesis of GSH. Thus, depletion of cysteine is an effective approach to trigger ferroptosis of cancer cells (Cheng et al., 2019). Dong et al. designed charge-transfer complexes nanoparticles for NIR-II PA imaging-guided PTT and ferroptosis (Ou et al., 2021). TMB could form charge-transfer complexes with TCNQ or F4TCNQ. After encapsulating these complexes with F127 and folic acid-capped PEG, the nanoparticles (TMB-TCNQ and TMB-F4TCNQ) were prepared. Under NIR-II laser irradiation, these nanoparticles exhibited both NIR-II PA signal and photothermal effect. On the other hand, the cyano groups of TCNQ and F4TCNQ could react with cysteine to form adducts which may deplete cysteine within cancer cells to induce ferroptosis (Figure 4A).

The ESR spectra demonstrated the formation of charge-transfer complexes between TMB and TCNQ or F4TCNQ. In addition, TMB had a higher charge transfer degree with F4TCNQ than TCNQ (72 vs. 48%) indicated by Raman spectra. Both TMB-TCNQ and TMB-F4TCNQ had strong NIR-II absorption. Under 1,060 nm laser irradiation, the solution temperature of TMB-TCNQ and TMB-F4TCNQ increased over time. Furthermore, TMB-F4TCNQ had a higher temperature increase than TMB-TCNQ (22.4 vs. 17.8°C), indicating the higher PCE of TMB-F4TCNQ. With treatment of cysteine, the absorption of TMB-F4TCNQ in the NIR region decreased with the increase of treatment time under 50 °C, which could be attributed to the reaction between F4TCNQ and cysteine. On the contrary, other antioxidants such as GSH, N-acetyl-l-cysteine (NAC) may not react with TMB-F4TCNQ and change its absorption. TMB-F4TCNQ could be internalized into 4T1 cells, and deplete GSH within cells. Meanwhile, the ROS and malondialdehyde level in 4T1 was elevated under TMB-F4TCNQ treatment, demonstrating that TMB-F4TCNQ could induce ferroptosis of 4T1 cells. Under 1,060 nm laser irradiation, the viability of both 4T1 and HeLa cells decreased with the increase of TMB-F4TCNQ concentration, and the IC50 for 4T1 and HeLa cells were calculated as 23 and 42 μg/ml (Figure 4C). By virtue of its strong NIR-II absorption, TMB-F4TCNQ was applied for *in vivo* NIR-II PA imaging. After injection, the NIR-II PA signal in tumor region of mice was significantly higher than that before injection, and such signal reached maximum at 4 h post-injection (Figure 4D). On the other hand, the tumor temperature could increase up to 53.6°C within 5 min under laser irradiation for TMB-F4TCNQ-injected mice. In addition, the tumor growth could be totally inhibited after treatment of TMB-F4TCNQ with laser irradiation, proving the anticancer efficacy of photothermal and ferroptosis combination therapy.

In summary, the above studies showed the examples of small molecule dyes for NIR-II PA imaging-guided combination therapy. Anticancer drugs, metal ions and cysteine responsive molecules were need to be introduced into the nanosystems to achieve the capability of combination therapy. Compared with the phototheranostic nanosystems discussed in the previous section, combination therapeutic nanosystems had much more complicated compositions. Although such feature made them still far from clinical translation at this stage, these studies expanded the biological applications of NIR-II PA imaging-based nanotheranostics and provided new horizons for combination therapy.

## 5 Discussion

We herein summarize recent advances in NIR-II PA imaging-guided phototheranostic nanosystems based on small molecule dyes. Owing to the high tissue penetration depth of NIR-II laser, NIR-II PA imaging has a remarkably high imaging depth and is suitable for tumor diagnosis. Compared with large molecules such as semiconducting polymers, small molecule dyes have relatively shorter metabolic time, which are more promise in the clinical translation. Until now, several small molecule dye-based nanotheranostics have been developed for NIR-II PA imaging-related applications. Cell membrane can be coated onto them to improve their tumor targeting capability for NIR-II PA imaging and PTT. By using surfactant-stripped approach, high concentration nanosystems can be prepared *via* removing excess F127 for NIR-II PA imaging and imaging-guided photothermal/chemo combination therapy. Besides chemotherapy, other therapeutic modalities including CDT and ferroptosis may be introduced into these nanosystems to further enrich their therapeutic function. These studies demonstrate the feasibility of small molecule dye-based nanosystems for the applications in NIR-II PA imaging and cancer therapy.

As discussed before, the small molecule dye-based NIR-II PA contrast agents can be classified into three types: NIR-II-absorbing molecules, metal complexes and charge-transfer complexes. Although all of them show satisfactory NIR-II PA imaging effect, their potential in such field is different. Metal complexes have very broad absorption spectrum. However, their absorption coefficient in the NIR-II region is relatively low [44] Meanwhile, such complexes are usually 3D networks and cannot be regarded as “small molecule” strictly. Charge-transfer complexes may have very long absorption wavelengths. However, at least two compounds are required to form such complexes *via* π-π stacking interaction, making them difficult to be prepared. (Ou et al., 2021). In addition, such interactions are readily to be destructed compared with covalent interaction. NIR-II-absorbing molecules are the most promising contrast agents among all these three types. They have strong NIR-II absorption with stable chemical structures. The only issue for NIR-II-absorbing molecules is the photostability, as some of them are cyanine derivatives which part of them are reported to have poor photostability (Zhao et al., 2019). Apart from these three types of agents, D-A-D type oligomers are also promising NIR-II PA contrast agents. They have a much better photostability than cyanine derivatives and are more facile to be synthesized (Yin et al., 2021b), which can be one of the future directions in the development of NIR-II PA contrast agents.

On the other hand, some other aspects can also be improved to promote the applications of these small molecule dyes in cancer theranostics. Until now, most reported small molecule dyes for NIR-II PA imaging are hydrophobic, which need to be transferred into nanoparticles to increase their water solubility for biological applications. Such nanoparticles are mostly micelles and the cargoes within them may suffer from the issue of burst release during circulation (Xie et al., 2017; Xie et al., 2020b). Modifying hydrophobic small molecule dyes with hydrophilic moieties to obtain amphiphilic dyes which can self-assemble in the aqueous solution is a feasible approach to address such issue. On the other hand, although small molecule dyes-formed nanoparticles have relatively faster clearance rate than polymers-based nanoparticles, they are still cleared out of body *via* hepatobiliary metabolism. Compared with hepatobiliary metabolism, renal clearance is a faster metabolism pathway which can minimized the long-term toxicity of materials (Huang et al., 2019c). Designing water-soluble small molecule dyes with the capability of renal clearance for NIR-II PA imaging may become one of the future directions.
